# Cigarette smoking and risk of severe infectious respiratory diseases in UK adults: 12-year follow-up of UK biobank

**DOI:** 10.1093/pubmed/fdad090

**Published:** 2023-06-22

**Authors:** Luke J McGeoch, Stephanie Ross, M Sofia Massa, Sarah Lewington, Robert Clarke

**Affiliations:** Clinical Trial Service Unit and Epidemiological Studies Unit, Nuffield Department of Population Health, University of Oxford, Oxford OX3 7LF, UK; Clinical Trial Service Unit and Epidemiological Studies Unit, Nuffield Department of Population Health, University of Oxford, Oxford OX3 7LF, UK; Clinical Trial Service Unit and Epidemiological Studies Unit, Nuffield Department of Population Health, University of Oxford, Oxford OX3 7LF, UK; Clinical Trial Service Unit and Epidemiological Studies Unit, Nuffield Department of Population Health, University of Oxford, Oxford OX3 7LF, UK; MRC Population Health Research Unit, Nuffield Department of Population Health, University of Oxford, Oxford OX3 7LF, UK; Clinical Trial Service Unit and Epidemiological Studies Unit, Nuffield Department of Population Health, University of Oxford, Oxford OX3 7LF, UK; MRC Population Health Research Unit, Nuffield Department of Population Health, University of Oxford, Oxford OX3 7LF, UK

**Keywords:** health intelligence, morbidity and mortality, smoking

## Abstract

**Background:**

The relevance of tobacco smoking for infectious respiratory diseases (IRD) is uncertain. We investigated the associations of cigarette smoking with severe IRD resulting in hospitalization or death in UK adults.

**Methods:**

We conducted a prospective study of cigarette smoking and risk of severe IRD in UK Biobank. The outcomes included pneumonia, other acute lower respiratory tract infections (OA-LRTI) and influenza. Multivariable Cox regression analyses were used to estimate hazard ratios (HRs) of severe IRD associated with smoking habits after adjusting for confounding factors.

**Results:**

Among 341 352 participants with no prior history of major chronic diseases, there were 12 384 incident cases with pneumonia, 7054 with OA-LRTI and 795 with influenza during a 12-year follow-up. Compared with non-smokers, current smoking was associated with ⁓2-fold higher rates of severe IRD (HR 2.40 [2.27–2.53] for pneumonia, 1.99 [1.84–2.14] for OA-LRTI and 1.82 [95% confidence interval: 1.47–2.24] for influenza). Incidence of all severe IRDs were positively associated with amount of cigarettes smoked. The HRs for each IRD (except influenza) also declined with increasing duration since quitting.

**Conclusions:**

Current cigarette smoking was positively associated with higher rates of IRD and the findings extend indications for tobacco control measures and vaccination of current smokers for prevention of severe IRD.

## Introduction

Infectious respiratory diseases (IRDs) account for a substantial proportion of deaths worldwide and were the second leading cause of death in low-income countries in 2019.^1^ IRDs are the leading infectious cause of death in Europe, and 90% of these deaths occur in older people.^2^ Pneumococcal pneumonia and influenza were the leading causes of fatal IRD in older people worldwide prior to the COVID-19 pandemic.^3^ The World Health Organization estimated that COVID-19 accounted for ⁓15 million deaths between 1 January 2020 and 31 December 2021.^4^

Previous observational studies reported associations of cigarette smoking with higher risks of pneumonia, influenza and tuberculosis.^6–8^ Some studies reported positive associations of current smoking with COVID-19,^8^ but others had reported null or inverse associations.^9^ However, most of the available evidence on associations of current smoking with severe IRD involved retrospective or prospective studies of participants without excluding individuals with prevalent diseases, and hence, such studies were unable to fully exclude the effects of reverse causality bias or of confounding by other factors. Few studies investigated associations with the amount of cigarettes smoked or the impact of smoking cessation with risks of IRD.

The present study examined the associations of cigarette smoking with incident severe IRD in UK Biobank after adjusting for confounding factors and controlling for reverse causation, including associations of amount of cigarettes smoked with severe IRD; and possible reversibility of such associations after quitting smoking.

## Methods

### Study population

UK Biobank is a population-based prospective cohort study of 502 616 UK adults that were recruited in 2006–2010. Postal invitations were sent to 9.2 million men and women aged 40–69 years who were living within 25 km of one of 22 study assessment centers and registered with the National Health Service in England, Scotland or Wales.^10^ The present study included information on smoking habits and covariates collected using validated touchscreen-administered questionnaires at enrolment.^10^ The UK Biobank study obtained ethical approval from the National Information Governance Board for Health and Social Care and the National Health Service North West Center for Research Ethics Committee (reference 11/NW/0382). The present analysis of the UK Biobank was conducted under application number 31461.

### Assessment of smoking status, covariates and prior diseases

Participants were classified as current cigarette smokers, former smokers or never smokers. Smoking habit was derived from participant responses to study questions: ‘Do you smoke now’ and ‘In the past, how often have you smoked tobacco’.^10^ Individuals who reported smoking cessation within the 12 months preceding recruitment were also considered to be smokers.^11^ Individuals who reported primarily smoking a pipe or cigars were excluded. The amount of cigarettes smoked daily were classified as 0–12, 13–22 or ≥ 23 cigarettes daily and duration (in years) since quitting among former smokers was classified as 1–15, 16–30 or ≥ 30 years. Repeat measures of smoking status were collected in non-randomly selected population resurveys between 2012 and 2021.^12^

Alcohol consumption was recorded using standard questions. Highest levels of educational attainment were categorized using the International Standard Classification of Education qualifications.^13^ Townsend deprivation index was derived from postcodes of main residence using data on unemployment, car and home ownership and household overcrowding.^14^ Self-reported information on prior diseases at enrolment in UK Biobank included IRD, chronic respiratory disease, major cardiovascular disease, type 2 diabetes and cancer (excluding non-melanoma skin cancer). Participants with any of these diseases were excluded to minimize risk of reverse causation, since such comorbidities may have resulted in changes in smoking habit.^15^ In the analyses of COVID-19, participants with hospitalizations during follow-up to 1 January 2020 that were coded with an *International Classification of Diseases tenth revision* (ICD-10) or *ninth revision* (ICD-9) diagnostic codes for any of these prior diseases (Supplementary Table 1) were also excluded.

### Hospitalization and mortality data

The IRD outcomes involved first IRD events, defined as any first recorded hospitalization or death occurring during follow-up prior to 25 February 2021, that included a main (underlying) or secondary diagnosis of severe IRD. Diagnoses were ascertained using nationally representative hospitalizations and mortality datasets linked to UK Biobank, in which diseases were coded using ICD-10 or ICD-9 diagnostic codes. Hospitalizations were recorded using the Hospital Episode Statistics (England and Wales) and Scottish Morbidity Records (Scotland). The IRD diagnoses included influenza (ICD-10 J09-J11; ICD-9487-488), other acute lower respiratory tract infection (OA-LRTI, ICD-10; J20-J22; ICD-9466), pneumonia (ICD-10 J12-J18; ICD-9480-486) and COVID-19 (ICD-10 U07). Previous studies had demonstrated the validity of ICD-10 codes for recording hospitalizations attributable to pneumonia, influenza and COVID-19.^16^^–^^20^ The OA-LRTI outcome included diagnostic codes for acute bronchitis, acute bronchiolitis or ‘unspecified acute lower respiratory infection’.

### Statistical analysis

To assess consistency in smoking status during follow-up, the percentages of participants in each smoking category at baseline were compared with those recorded at resurveys during follow-up (<5 years, 5 to <10 years, ≥10 years) after stratifying by baseline smoking habits.

Cox proportional hazards models were used to estimate hazard ratios (HRs) and 95% confidence intervals (95% confidence interval [CI]) for the associations of cigarette smoking status with IRD, with never smoking as the reference group. COVID-19 analyses were left truncated at 1 January 2020. Participants who did not experience any disease outcomes were censored at the date of death, loss to follow-up or end of follow-up, whichever occurred first.

The minimally adjusted models were adjusted for age, sex and ethnicity. Additional analyses included adjustment for alcohol consumption, body mass index (BMI), Townsend deprivation index and educational attainment. The proportional hazards assumption was tested by fitting smoking status and each covariate as time-varying covariates (and was not violated for any variables). Sensitivity analyses involved: (i) outcomes restricted to hospitalizations and deaths with the relevant IRD codes as the main (underlying) cause and (ii) excluding outcomes occurring during the first 2 years of follow-up.

The associations of daily amount of cigarettes smoked with severe IRD among smokers, and duration since quitting smoking among former smokers, were assessed by fitting these exposures as categorical variables with never smoking as the reference category. Group-specific variances were estimated in Cox regression analyses, to allow comparisons between any two categories in addition to a single reference group.^19^ All analyses were conducted using Stata v16.1.

### Role of the funding source

The funders had no role in the study design, data collection, data analysis, data interpretation or writing of the report.

## Results

### Baseline characteristics of study participants

A total of 502 460 UK Biobank participants provided consent for follow-up prior to 25 February 2021. The analyses successively excluded 2949 participants due to missing data on smoking status, 7558 due to pipe or cigar smoking, 141 255 due to prior diseases (9194 with prior severe IRD, 64 421 with non-IRD, 34 548 with cancer, 25 764 with diabetes and 30 797 with cardiovascular disease) and 9346 due to incomplete data on covariates (6036 due to missing education: Supplementary Fig. 1). After these exclusions, the present analyses involved a total of 341 352 (67.9%) individuals, among whom 18 406 had severe IRD, 9626 died from other causes and 858 (0.3%) were lost to follow-up.

Table 1 shows selected characteristics of the study participants at baseline. Overall, the median age was 57 years, 56% were female and 95% had white ethnicity. A total of 197 153 (57.8%) were never smokers, 108 974 (31.9%) were former smokers and 35 225 (10.3%) were current cigarette smokers. Compared with never and former smokers, current smokers were younger, less likely to be female, more deprived and less highly educated. Compared with current smokers and never smokers, former smokers were more likely to be white, have a higher BMI and consumed more alcohol. The median duration of follow-up was 12 years for all participants and 14 months for the subset with COVID-19 analyses.

**Table 1:** Baseline characteristics of study participants, by smoking status

	Never smoking	Former smoking	Current smoking	Total
**Number of participants, *n* (%)**	197 153 (57.8)	108 974 (31.9)	35 225 (10.3)	341 352 (100.0)
**Age, median (IQR)**	56 (49, 62)	59 (52, 63)	53 (47, 60)	57 (49, 62)
**Female, *n* (%)**	117 244 (59.5)	57 469 (52.7)	16 991 (48.2)	191 704 (56.2)
**White, *n* (%)**	184 990 (93.8)	106 000 (97.3)	33 092 (93.9)	324 082 (94.9)
**≥3 alcoholic drinks/week, *n* (%)**	77 023 (39.1)	58 917 (54.1)	16 430 (46.6)	152 370 (44.6)
**BMI (kg/m** ^**2**^**), median (IQR)**	26.2 (23.7, 29.2)	26.9 (24.4, 29.8)	26.3 (23.7, 29.2)	26.4 (23.9. 29.4)
**High educational attainment, *n* (%)** ^a^	73 297 (37.2)	34 404 (31.6)	8714 (24.7)	116 415 (34.1)
**Townsend index, median (IQR)** ^b^	−2.46 (−3.81, −0.17)	−2.20 (−3.66, 0.30)	−0.63 (−2.89, 2.52)	−2.25 (−3.70, 0.28)

^a^Defined as completion of a university or college degree.

^b^Higher (less negative or more positive) scores indicate greater deprivation.

### Changes in smoking status during follow-up

Resurveys on smoking status were available for 51 365 (15.0%) non-randomly selected participants at resurveys, including 13 198 participants who attended resurveys <5 years after baseline, 25 774 participants between 5 and 9 years after baseline and 12 393 participants at 10 or more years after baseline (Table 2). Across the three resurvey periods, the proportion of current smokers at baseline that reported continuing to smoke declined progressively (63.3%, 49.7%, 37.4%, respectively) during follow-up. Thus, the true associations between current cigarette smoking and rate of severe IRD are likely to be stronger than those classified at baseline. By contrast, ⁓90% of former smokers at baseline and 98% of never smokers at baseline reported the same smoking status during all three follow-up periods.

**Table 2:** Distribution of never, former and current smokers at successive resurveys, stratified by smoking status reported at baseline (*n* = 51 365 participants)

Duration of follow-up and smoking status at resurvey, *n* (%)^a^	Smoking status at baseline		
	Never smoking	Former smoking	Current smoking
**<5 years**			
Never smoking	8113 (98.4)	371 (8.7)	18 (2.6)
Former smoking	126 (1.5)	3807 (89.7)	241 (34.1)
Current smoking	7 (0.1)	68 (1.6)	447 (63.3)
**5 to <10 years**			
Never smoking	15 793 (98.3)	723 (9.0)	40 (2.3)
Former smoking	261 (1.6)	7133 (89.2)	819 (48.0)
Current smoking	14 (0.1)	142 (1.8)	849 (49.7)
**≥10 years**			
Never smoking	7507 (98.2)	377 (9.5)	20 (2.5)
Former smoking	129 (1.7)	3534 (89.2)	474 (60.1)
Current smoking	7 (0.1)	50 (1.3)	295 (37.4)

^a^Repeat ascertainment of smoking status was undertaken in sub-samples of the original participant cohort that underwent resurveys between 2012 and 2021. Column percentages shown.

### Associations of current smoking with rates of severe IRD

**Fig. 1:**
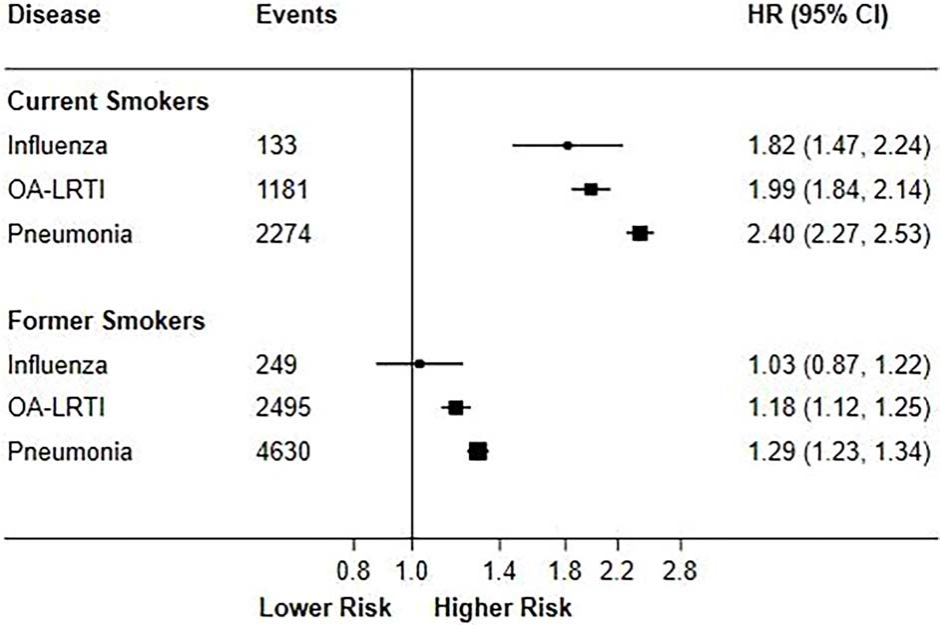
HRs (95% CI) of severe IRD for current smokers and former smokers versus never smokers. An event was defined as a hospitalization or mortality including a primary or secondary diagnosis of the relevant IRD. Among never smokers, there were 413 influenza events, 3378 OA-LRTI events and 5480 pneumonia events. HRs were adjusted for age, sex, ethnicity, alcohol consumption, body mass index, educational attainment and Townsend deprivation index. Fully adjusted HRs are shown as solid squares. The area of each square is inversely proportional to the standard error of the HR. 95% CIs are shown as horizontal black lines.

A total 795 first incident influenza cases, 7054 OA-LRTI cases and 12 384 pneumonia cases were recorded during follow-up. Fig. 1 shows the associations of current smoking and former smoking with rates of severe IRD after adjusting for all covariates (see Supplementary Fig. 2 for effects of sequential adjustment). Current cigarette smoking was associated with about a 2-fold higher rate of severe IRD (HR [95%CI]: 1.82 [1.47–2.24] for influenza, 1.99 [1.84–2.14] for OA-LRTI and 2.40 [2.27–2.53] for pneumonia). Former smoking was associated with 20–30% higher risks of severe IRD (HR [95%CI]: 1.18 [1.12–1.25] for OA-LRTI vs 1.29 [1.23–1.34] for pneumonia) but was unrelated with risks of influenza (1.03 [0.87–1.22]).

### Dose-response associations with amount of cigarettes smoked daily

**Fig. 2:**
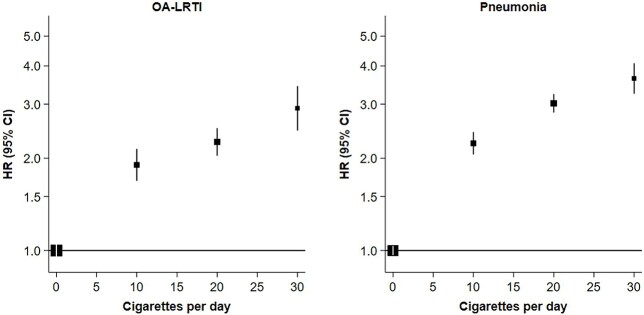
HRs (95%CI) of severe IRD for current smokers versus never smokers, by the amount of cigarettes smoked daily. The number of cigarettes smoked per day among current smokers, reported at recruitment, was categorized as 1–12, 13–22 or ≥23. Cox survival models were adjusted for age, sex, ethnicity, alcohol consumption, body mass index, educational attainment and Townsend deprivation index. Participants with existing IRD, chronic respiratory disease, cardiovascular disease, diabetes or cancer at baseline were excluded from the analyses. Symbols and conventions as in Fig. 1.

Data on the daily amount of cigarettes smoked at enrolment were available for 36 160 current smokers. A total of 14 711 (40.7%) participants reported smoking 1–12 cigarettes, 16 328 (45.2%) reported smoking 13–22 cigarettes and 5121 (14.2%) reported smoking ≥23 cigarettes daily. Compared to never smoking, the HRs for OA-LRTI increased progressively with amount of cigarettes smoked daily from 1.90 [1.67–2.16] for those smoking 1–12 cigarettes to 2.26 [2.08–2.53] for those smoking 13–22 cigarettes and 2.91 [2.45–3.46] for those smoking ≥23 cigarettes daily. Likewise, the HRs for pneumonia were 2.24 [2.04–2.45], 3.02 [2.80–3.06] and 3.64 [3.23–4.10] for the latter categories of cigarettes smoked, respectively (Fig. 2). There were insufficient numbers of influenza cases to estimate results reliably by the amount of cigarettes smoked.

### Comparing strength of association with increasing duration since quitting smoking

**Fig. 3:**
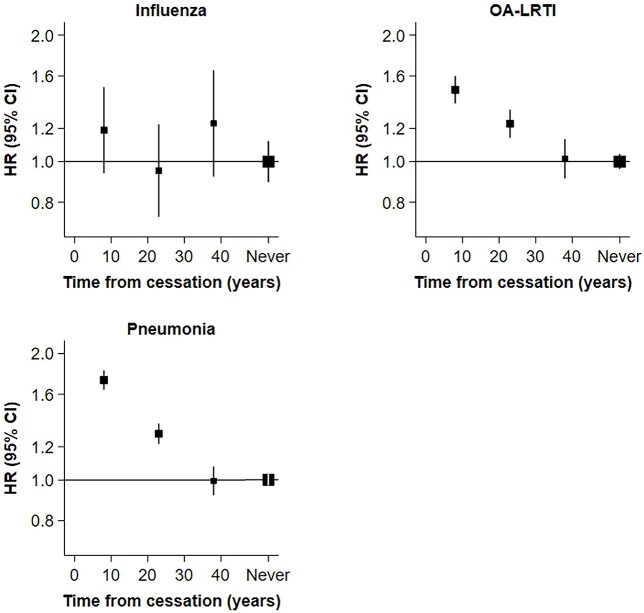
HRs of (95%CI) of severe IRD types for former versus never smokers, by time from cessation of smoking. The interval time in years from smoking cessation among former smokers was defined from study baseline and categorized as 1–15 years, 16–30 years or ≥31 years. Cox survival models were adjusted for age, sex, ethnicity, alcohol consumption, body mass index, educational attainment and Townsend deprivation index. Solid squares represent fully adjusted estimates of HRs. Symbols and conventions as in Fig. 1.

Data on duration (in years) since quitting smoking were available for 117 061 former smokers. A total of 48 558 (41.5%) reported cessation 1–15 years before baseline, 45 284 (38.7%) 16–30 years before baseline and 23 219 (19.8%) ≥31 years before baseline. Fig. 3 shows a progressive decline in rates of severe IRD with increasing intervals from quitting smoking for OA-LRTI (HR [95%CI]: 1.48 [1.36–1.61] at 0–16 years, 1.23 [1.13–1.34] at 16–30 years, 1.01 [0.90–1.14] at ≥31 years). The findings were similar for pneumonia. For influenza, there were no discernible differences in the rate irrespective of the duration since quitting smoking, though there is still some uncertainty about these estimates.

### Associations with COVID-19

The associations of smoking with COVID-19 in the subset of UK Biobank participants are presented in the Supplementary material. A total of 264 748 (52.7%) participants in the UK Biobank were included in the present analyses, but 220 721 participants were excluded due to prior disease and 6493 due to missing data on covariates. The baseline characteristics for this subset are shown in Supplementary Table 2. A total of 684 COVID-19 cases occurred during follow-up. Both current smoking (1.27 [0.99–1.64]) and former smoking (1.30 [1.10–1.54]) were modestly associated with higher risks of severe COVID-19 (Supplementary Fig. 2), but the number of cases among current smokers was limited.

**Fig. 4:**
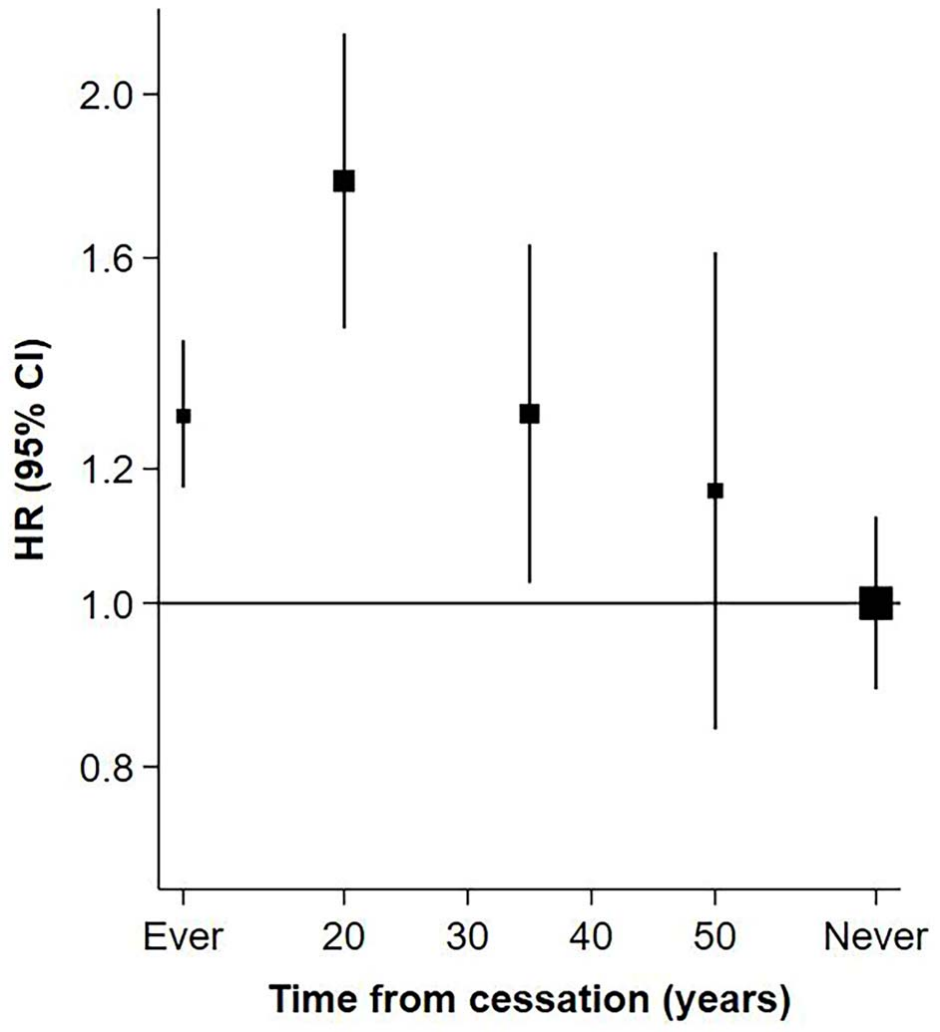
Rate of severe COVID-19 for former smokers versus ever smokers and never smokers, by time from cessation of smoking. The interval in years from smoking cessation among former smokers was defined from study baseline and categorized as 13–27 years, 28–42 years, ≥43 years. Every smokers included both current and former smokers. Cox regression models were adjusted for age, sex, ethnicity, alcohol consumption, body mass index, educational attainment and Townsend deprivation index. Symbols and conventions as in Fig. 1.

Given left censoring to January 2020 in COVID-19 analyses, the intervals from baseline at which former smokers had quit smoking were 13–27 years, 28–42 years and ≥43 years. There was a progressive decline in HRs with increasing intervals from cessation, from a HR of 1.78 [1.41–2.25] at 13–27 years, to 1.29 [1.00–1.68] at 28–42 years and 1.16 [0.82–1.64] at ≥43 years (Fig. 4).

### Sensitivity analyses

Hospitalizations and deaths in which IRDs were coded as the main diagnosis accounted for over half of all IRD cases (COVID-19: 75.1%; influenza: 71.9%; OA-LRTI: 56.8%; pneumonia: 58.8%). Associations of smoking status with rate of severe IRD were directionally consistent and comparable in magnitude to those observed in the main analyses (Supplementary Fig. 3). After excluding cases occurring during the first 2 years of follow-up, associations of smoking status with severe IRD did not differ from those observed in the main analyses (Supplementary Fig. 4).

## Discussion

This prospective study of ⁓341 000 UK adults demonstrated that current smoking was associated with 2-fold higher incidence of severe influenza, OA-LRTI and pneumonia compared with never smoking. Current smoking was also modestly associated with higher risks of COVID-19. The rates of severe influenza and pneumonia were related to the amount of cigarettes smoked daily, with ⁓50% higher rates for those smoking ≥23 cigarettes versus 1–12 cigarettes daily. Former smoking was less strongly associated with the rates of severe COVID-19, OA-LRTI and pneumonia, though the rates still exceeded 1.40 in participants who had quit smoking 1–15 years prior to enrolment, even for COVID-19 in which the first cases occurred over a decade later and declined with increasing duration since quitting smoking. The associations were independent of confounders and persisted following exclusion of cases recorded during early follow-up, suggesting that the associations were unlikely to be explained by confounding or reverse causality.

The findings that current smoking and former smoking were both associated with higher rates of pneumonia-related hospitalizations or mortality are consistent with results from previous smaller studies.^5^^,^^20^ However, the available evidence on the associations of current cigarette smoking with influenza are limited. Population-based prospective studies have previously reported associations between either smoking or former smoking and COVID-19 hospitalization or mortality.^9^^,^^21^ The findings of the present study demonstrated that former smoking was associated with higher rates of severe COVID-19, but there were insufficient cases of severe COVID-19 among current smokers to provide conclusive evidence of associations on current smoking and COVID-19 (as the lower 95%CI for these associations were 0.99). Secondly, current smokers may have been more likely than former or never smokers to adhere to COVID-19 isolation measures due to perceived higher risks of adverse health outcomes, which might underestimate any associations with smoking. Thirdly, previous reports of null or protective associations between current smoking and COVID-19 may have been constrained by variable adjustment for mediating factors,^9^ misclassification of smoking status^22^ or collider bias.^23^

A previous study investigated both the observational and genetically instrumented associations between smoking and risk of hospital admissions or deaths from COVID-19 to 18 August 2020.^24^ The latter study reported that cigarette smoking was associated with higher odds of hospitalization from COVID-19 (OR 1.80 [95%CI: 1.26–2.29] for current smoking and 1.31 [1.14–1.50] for former smoking) and with COVID-19 mortality (4.89 [3.41–7.00] for current smoking and 1.60 [1.29–1.97] for former smoking, respectively).

The observed associations between amount of daily cigarette consumption and rates of severe OA-LRTI and pneumonia were consistent with previous reports for pneumonia hospitalization and mortality.^20^^,^^25^ The progressive decline in rates of severe COVID-19, OA-LRTI and pneumonia with increasing duration since quitting smoking are also consistent with a progressive reduction in the risk of smoking-related diseases among individuals who quit smoking in such studies,^26^ including a decline in the risks of all-cause, respiratory and vascular mortality (with comparable risks for those who discontinued smoking ≥20 years earlier with never smokers).^26^

The possible mechanisms linking associations between smoking and IRD, include IRD of viral etiology (severe influenza and COVID-19) in addition to IRD of bacterial etiology (pneumonia and OA-LRTI) and may reflect a greater susceptibility to infection, impaired immune responses to infection, exacerbation of disease pathways or a combination of all such mechanisms.^27^ Importantly, many of these changes appear to be reversible with smoking cessation.^27^

This report highlights the importance of cigarette smoking as a risk factor for severe IRD and could extend the indications for tobacco control measures to prevent initiation of smoking to include severe IRD.^28^ Effective smoking cessation interventions have been reported among individuals diagnosed with IRDs and hospitalizations during which IRD was diagnosed afford an important opportunity for promotion of smoking cessation.^29^^,^^30^ Effective vaccines have also been developed for the prevention of pneumococcal pneumonia, influenza and COVID-19.^31–33^ In the UK, routine pneumococcal and influenza vaccinations are recommended for individuals with chronic obstructive pulmonary disease (COPD), but not for smokers without COPD.^34^^,^^35^ The US Center for Disease Control recommends pneumococcal vaccination for all adult smokers.^36^ The present findings, together with those of previous studies, could provide support for recommendations for seasonal influenza, pneumococcal and potentially COVID-19 vaccination to all adult smokers.

This study has several strengths. We assessed the impact of smoking habits on severe IRDs in a prolonged follow-up of a large prospective study. Questions on smoking status were well defined, limiting misclassification between current and former smoking. Reliable linkage to hospitalization and mortality data, including deaths in the community, permitted long-term passive follow-up of clinical diagnoses, minimizing the risk of response and attrition biases. Access to highly complete data on a wide range of covariates minimized risk of residual confounding. One of the novel features of the present study was the ability to control for the effects of reverse causality on the associations of smoking with IRD outcomes. The study included an assessment of dose-response of IRD by amount of cigarettes smoked and the strength of association by duration since quitting smoking.

This study also had several limitations. Firstly, UK Biobank was constrained by selection bias (healthy volunteers) and had a low response rate of 5.5%. However, given the large size and heterogeneity of exposures, exposure-outcome associations should be generalizable to most of the UK population.^12^ The analyses relied on self-reported smoking habits, which could have resulted in some misclassification of smoking habits. Likewise, infectious disease status may have been non-differentially misclassified in some cases due to the use of ICD-10 codes corresponding to clinical rather than microbiological or radiographic diagnoses, or coding errors. However, previous studies have confirmed the validity for IRD hospitalizations.^18–20^ This study did not investigate the associations of cigarette smoking with minor IRD diagnosed in the community. Finally, a high proportion of smokers quit smoking during follow-up, and hence, the true strength of associations of continuing smoking with severe IRD may be even greater due to regression dilution. Finally, there were no data available on vaccination status for influenza, COVID-19 or pneumococcal vaccines.

The present study demonstrates strong positive associations between current cigarette smoking with severe influenza, OA-LRTI and pneumonia, which extend the indications for tobacco control measures.

## Supplementary Material

Supplemental_Material_Supplementary_Tables_and_Figures_fdad090

## Data Availability

UK Biobank data are available on application (www.ukbiobank.ac.uk/).

## Notes

**Luke J. McGeoch**, Specialty Registrar in Public Health

**Stephanie Ross**, Teaching and Research Fellow

**M Sofia Massa**, Senior Statistician

**Sarah Lewington**, Professor of Epidemiology and Medical Statistics

**Robert Clarke**, Professor of Epidemiology and Public Health Medicine
