# Supplementary material for: Cigarette smoking and risk of severe infectious respiratory diseases in UK adults: 12-year follow-up of UK biobank
Source: J Public Health (Oxf). 2023 Jun 22;45(4):e621–9. doi: 10.1093/pubmed/fdad090 (PMC10687597; doi:10.1093/pubmed/fdad090)
Supplement: Supplemental_Material_Supplementary_Tables_and_Figures_fdad090 [file supplemental_material_supplementary_tables_and_figures_fdad090.docx]

**Supplementary information for ‘Smoking and risk of severe infectious respiratory diseases in UK adults: a 12-year follow-up of UK Biobank’**

**Luke J. McGeoch, Stephanie Ross, M. Sofia Massa, Sarah Lewington, Robert Clarke**

Contents

[**Supplement**a**ry Table 1: ICD-10 and ICD-9 codes for prior diseases excluded in COVID-19 analyses.** 2](#_Toc108081666)

[**Supplementary Table 2: Baseline characteristics of study participants included in COVID-19 analyses by smoking status.** 3](#_Toc108081667)

[**Supplementary Figure 1: Number of UK Biobank participants excluded from the present study and reasons for exclusion.** 4](#_Toc108081668)

[**Supplementary Figure 2: Hazard ratios (95% CIs) for severe IRD comparing smoking and former smoking with never smoking following sequential adjustment for covariates.** 5](#_Toc108081669)

[**Supplementary Figure 3: Hazard ratios (95% CIs) for hospitalisation or mortality including IRD as the primary diagnosis comparing smoking and former smoking with never smoking.** 6](#_Toc108081670)

[**Supplementary Figure 4: Hazard ratios (95% CIs) for severe IRD comparing smoking and former smoking with never smoking following exclusion of the first two years of follow-up.** 7](#_Toc108081671)

# **Supplement**a**ry Table 1: ICD-10 and ICD-9 codes for prior diseases excluded in COVID-19 analyses.**

| **Disease Category** | **ICD-10** | **ICD-9** | **Diagnosis** |
| --- | --- | --- | --- |
| Infectious Respiratory Disease | A15-A19  J09-J11  J12-J18  J20-J22  J85-J86  U07 | 010-018  487-488  480-486  466  510, 511.1, 513 | Tuberculosis  Influenza  Pneumonia  Other acute lower respiratory tract infections  Abscess or pyothorax  COVID-19 |
| Chronic Respiratory Disease | J40-J47  J60-J70  J80-J84  J95.3  J96.1  J96.9  J98.2  J98.3  J99.0 | 490-496  500-508  515-517  518.5 | Chronic lower respiratory diseases  Lung diseases due to external agents  Other respiratory diseases principally affecting the interstitium  Chronic pulmonary insufficiency following surgery  Chronic respiratory failure  Respiratory failure, unspecified  Interstitial emphysema  Compensatory emphysema  Rheumatoid lung disease |
| Cardiovascular Disease | I05-I09  I11; I13.0-I13.2  I20-I25  I34-I37; I39.0-I39.4  I42-I43  I50  I60-162  I64  165-166  I71-I72 | 394-397  402, 4030, 404  410-414, 429.5-429.7  424  425  4280, 4282, 4283  430-432  433-434  441, 443.2 | Chronic rheumatic heart diseases  Hypertensive heart disease  Ischaemic heart diseases  Valve disorders  Cardiomyopathy  Heart failure  Intracranial haemorrhage  Stroke  Occlusion and stenosis of precerebral or cerebral arteries  Arterial aneurysm or dissection |
| Type II Diabetes Mellitus | E10-E14 | 249-250 | Diabetes mellitus |
| Cancer | C00-C43, C45-C97 | 140-172, 174-209 | Malignant neoplasms (excluding non-melanoma skin cancer) |
| *Abbreviation: ICD, international classification of diseases.* | | | |

# **Supplementary Table 2: Baseline characteristics of study participants included in COVID-19 analyses by smoking status.**

|  | **Never smoking** | **Former smoking** | **Current smoking** | **Total** |
| --- | --- | --- | --- | --- |
| **Number of participants, n (%)** | 158,594 (59.9) | 81,473 (30.8) | 24,681 (9.3) | 264,748 (100.0) |
| **Age, median (IQR)** | 55 (48, 61) | 57 (50, 63) | 52 (45, 59) | 55 (48, 61) |
| **Female, n (%)** | 96,309 (60.7) | 45,089 (55.3) | 12,321 (49.9) | 153,719 (58.1) |
| **White, n (%)** | 148,873 (93.9) | 79,178 (97.2) | 23,047 (93.4) | 251,098 (94.8) |
| **≥3 alcoholic drinks/week, n (%)** | 62,592 (39.5) | 44,528 (54.7) | 11,833 (47.9) | 118,953 (44.9) |
| **BMI (kg/m^2^), median (IQR)** | 26.0 (23.6, 28.9) | 26.6 (24.1, 29.4) | 26.1 (23.6, 29.0) | 26.2 (23.7, 29.1) |
| **High educational attainment, n (%)*** | 60,898 (38.4) | 27,273 (33.4) | 6,725 (27.3) | 94,896 (35.8) |
| **Townsend index, median (IQR)**^†^ | -2.48 (-3.83, -0.22) | -2.21 (-3.67, 0.25) | -0.77 (-2.94, 2.33) | -2.29 (-3.71, 0.18) |
| * Defined as completion of a university or college degree.  ^†^ Higher (less negative or more positive) scores indicate greater deprivation.  *Abbreviations: BMI, body mass index; COPD, chronic obstructive pulmonary disease.* | | | | |

# **Supplementary Figure 1: Number of UK Biobank participants excluded from the present study and reasons for exclusion.**


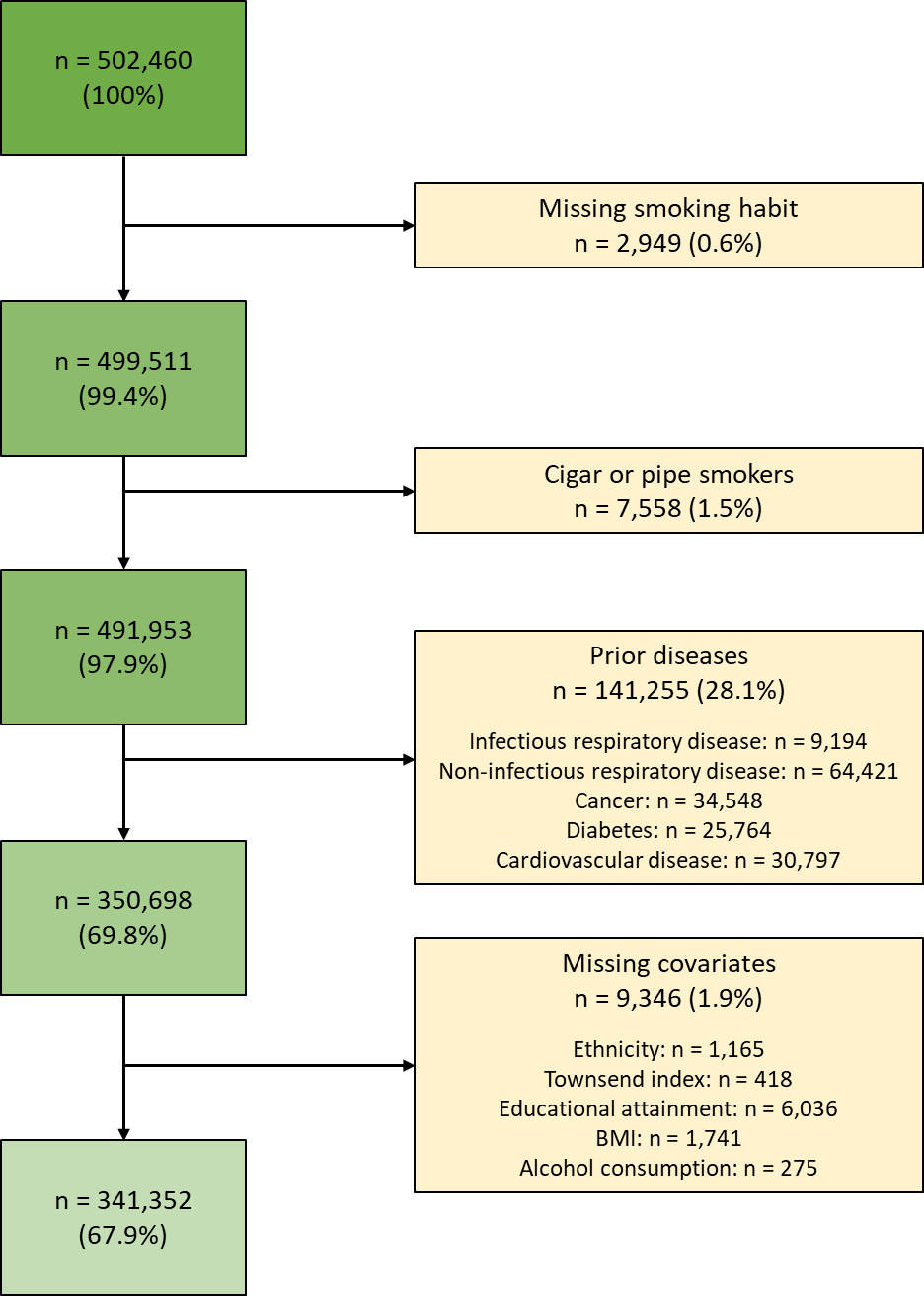


# **Supplementary Figure 2: Hazard ratios (95% CIs) for severe IRD comparing current and former smoking with never smoking following sequential adjustment for covariates.**


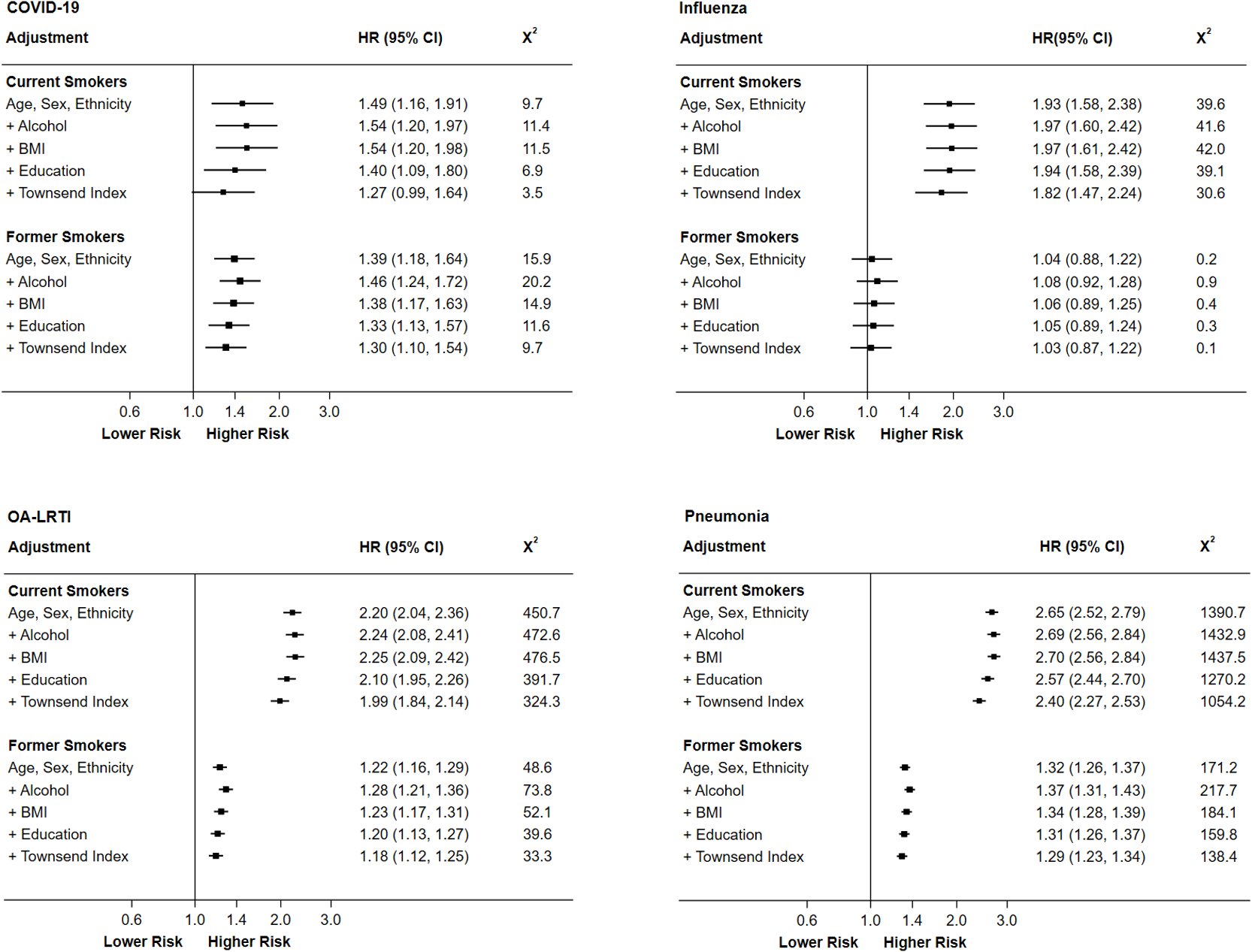


| Education was defined as high attainment (university or college degree), medium attainment (secondary educational or vocational qualification) or low attainment (none of the above). The Townsend deprivation index is a census-derived measure of socioeconomic status based on home ownership, car ownership, unemployment and houshold overcrowding. Participants with existing IRD, chronic respiratory disease, cardiovascular disease, diabetes or cancer were excluded from the analyses. HRs are shown as solid squares. The area of each square is inversely proportional to the standard error of the HR. 95% CIs are shown as horizontal black lines. χ^2^ values corresponding to smoking or former smoking have 1 degree of freedom and can be compared only within single smoking groups and diseases.  *Abbreviations: BMI, body mass index; CI, confidence interval; HR, hazard ratio; IRD, infectious respiratory disease; OA-LRTI other acute, lower respiratory tract infection.* |
| --- |

# **Supplementary Figure 3: Hazard ratios (95% CIs) for hospitalisation or mortality including IRD as the primary diagnosis comparing current smoking and former smoking with never smoking.**


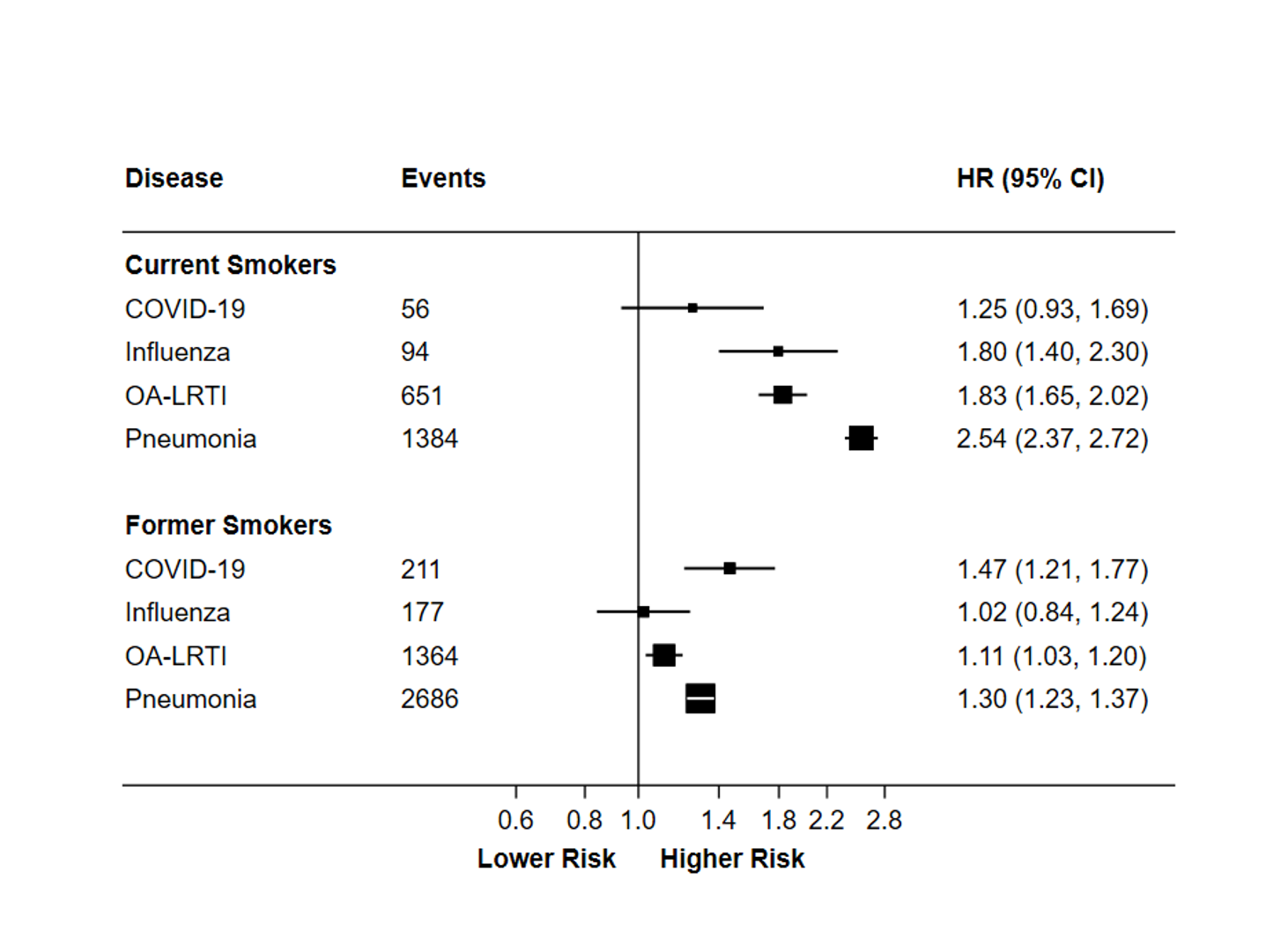


| An event was defined as a hospitalisation or mortality including a primary or secondary diagnosis of the relevant IRD. Among never smokers, there were 247 COVID-19 events, 301 influenza events, 1,993 OA-LRTI events and 3,216 pneumonia events. Prior diseases were identified based on participant self-report at baseline. HRs were adjusted for age, sex, ethnicity, alcohol consumption, body mass index, educational attainment, and Townsend deprivation index. Fully adjusted HRs are shown as solid squares. The area of each square is inversely proportional to the standard error of the HR. Horizontal black lines indicate floating 95% CIs, shown as a horizontal white line where otherwise concealed by the point estimate marker.  *Abbreviations: CI, confidence interval; HR, hazard ratio; IRD, infectious respiratory disease; OA-LRTI, other acute lower respiratory tract infection.* |
| --- |

# **Supplementary Figure 4: Hazard ratios (95% CIs) for severe IRD comparing current smoking and former smoking with never smoking following exclusion of the first two years of follow-up.**


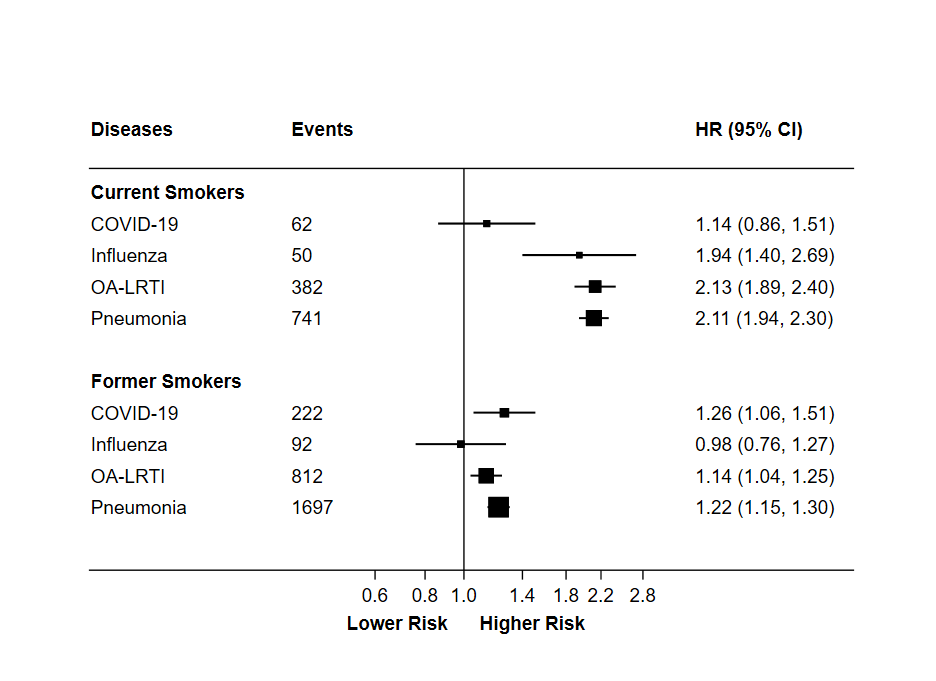


| An event was defined as a hospitalisation or mortality including a primary or secondary diagnosis of the relevant IRD. Among never smokers, there were 311 COVID-19 events, 167 influenza events, 1,228 OA-LRTI events and 2,328 pneumonia events. Prior diseases were identified based on participant self-report at baseline. HRs were adjusted for age, sex, ethnicity, alcohol consumption, body mass index, educational attainment, and Townsend deprivation index. Fully adjusted HRs are shown as solid squares. The area of each square is inversely proportional to the standard error of the HR. 95% CIs are shown as horizontal black lines.  *Abbreviations: CI, confidence interval; HR, hazard ratio; IRD, infectious respiratory disease; OA-LRTI, other acute lower respiratory tract infection.* |
| --- |
